# Gonadotropin‐releasing hormone agonist treatment and ischemic heart disease among female patients with breast cancer: A cohort study

**DOI:** 10.1002/cam4.5390

**Published:** 2022-10-28

**Authors:** Yi‐Sheng Chou, Chun‐Chieh Wang, Li‐Fei Hsu, Pei‐Hung Chuang, Chi‐Feng Cheng, Nai‐Hsin Li, Chu‐Chieh Chen, Chien‐Liang Chen, Yun‐Ju Lai, Yung‐Feng Yen

**Affiliations:** ^1^ School of Medicine National Yang Ming Chiao Tung University Taipei Taiwan; ^2^ Department of Hematology and Oncology Taipei City Hospital, Renai Branch Taipei Taiwan; ^3^ Division of Chest Medicine, Department of Internal Medicine Puli branch of Taichung Veterans General Hospital Taichung Taiwan; ^4^ College of Public Health National Taiwan University Taipei Taiwan; ^5^ Taipei Association of Health and Welfare Data Science Taipei Taiwan; ^6^ Department of General Surgery Taipei City Hospital, Renai Branch Taipei Taiwan; ^7^ Department of Health Care Management National Taipei University of Nursing and Health Sciences Taipei Taiwan; ^8^ Department of Economics National Chi Nan University Nantou Taiwan; ^9^ Division of Endocrinology and Metabolism, Department of Internal Medicine Puli Branch of Taichung Veterans General Hospital Nantou Taiwan; ^10^ Department of Exercise Health Science National Taiwan University of Sport Taichung Taiwan; ^11^ Institute of Public Health National Yang Ming Chiao Tung University Taipei Taiwan; ^12^ Section of Infectious Diseases Taipei City Hospital, Yangming Branch Taipei Taiwan; ^13^ Department of Education and Research Taipei City Hospital Taipei Taiwan

**Keywords:** breast neoplasms, cardiovascular diseases, gonadotropin‐releasing hormone, heart disease risk factors, myocardial ischemia

## Abstract

**Background:**

The risk of ischemic heart disease (IHD) due to the impact of gonadotropin‐releasing hormone (GnRH) agonists among female patients with breast cancer remains a controversy.

**Methods:**

Information from the Registry for Catastrophic Illness, the National Health Insurance Research Database (NHIRD), and the Death Registry Database in Taiwan were analyzed. Female patients with breast cancer were selected from the Registry for Catastrophic Illness from January 1, 2000, to December 31, 2018. All the breast cancer patients were followed until new‐onset IHD diagnosis, death, or December 31, 2018. A Kaplan–Meier survival curve was drawn to show the difference between patients treated with and without GnRH agonists. The Cox regression analysis was used to investigate the effects of GnRH agonists and the incidence of IHD.

**Results:**

A total of 172,850 female patients with breast cancer were recognized with a mean age of 52.6 years. Among them, 6071(3.5%) had received GnRH agonist therapy. Kaplan–Meier survival curves showed a significant difference between patients with and without GnRH therapy (log‐rank *p* < 0.0001). Patients who received GnRH therapy had a significantly decreased risk of developing IHD than those without GnRH therapy (HR = 0.18; 95% CI = 0.14–0.23). After adjusting for age, treatment, and comorbidity, patients who received GnRH therapy still had a significantly lower risk of developing IHD (AHR = 0.5, 95% CI = 0.39–0.64).

**Conclusion:**

The study showed that the use of GnRH agonists for breast cancer treatment was significantly associated with a reduced risk of IHD. Further research is required to investigate the possible protective effect of GnRH on IHD.

## Graphical abstract

For female patients with breast cancer, the use of GnRH agonists was significantly associated with a reduced risk of IHD. After adjusting for age, treatment, and comorbidity, patients who received GnRH therapy had a significantly lower risk of developing IHD (AHR=0.5, 95% CI=0.39‐0.64). GnRH agonists were significantly associated with a lower risk of incident IHD in all the subgroups, except in those with CKD or COPD, respectively.

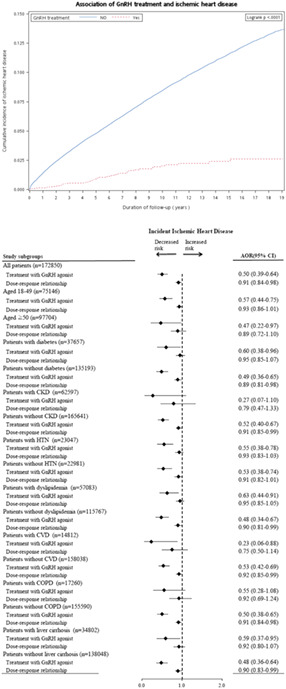

## 1 INTRODUCTION

Breast cancer is the most common cancer among females worldwide, accounting for 25.4% of total women's cancer, with more than two million newly diagnosed cases.^1^ In Asia, female patients with breast cancer were younger compared with patients from Western countries. Luminal histology subtypes were also more predominate among patients in Western countries.^2^ For patients with premenopausal or perimenopause endocrine positive breast cancer, gonadotropin‐releasing hormone (GnRH) agonists are increasingly administered in combination with tamoxifen^3^ or cyclin‐dependent kinase 4/6 inhibitor^4^, ^5^ in the adjuvant or metastatic settings. GnRH agonists inhibit the pituitary GnRH receptors and suppress the downstream effects of follicle‐stimulating hormone (FSH) and luteinizing hormone (LH), resulting in decreased estrogen production in premenopausal ovaries.^6^

Previous studies have shown diverse results regarding the effects of GnRH agonists on the cardiovascular system for hormone‐dependent cancer management. A previous animal study showed that GnRH agonists may be associated with atherosclerotic effects.^7^ Several observational studies showed that GnRH agonists were related to increased cardiovascular disease risk in patients with prostate cancer.^8^, ^9^, ^10^ However, a meta‐analysis of randomized trials reported no significant associations between GnRH agonists and the risk of cardiovascular disease.^11^ Most evidence suggesting an association between GnRH agonists and cardiovascular disease for male patients with prostate cancer came from population‐based studies.^8^, ^9^, ^12^, ^13^ Several meta‐analyses of observational studies disclosed that GnRH agonists were related to an increased incidence of non‐fatal cardiovascular disease.^14^, ^15^ Whether or not GnRH agonists are associated with an excess risk of cardiovascular morbidity remains a highly controversial question.^11^

To the best of our knowledge, limited literature addressing the associations between GnRH agonists and the risk of cardiovascular disease in patients with breast cancer is available. Therefore, this study intended to determine the relationship between GnRH agonists and the risk of IHD in female breast cancers.

## 2 METHODS

### 2.1 Data source

Data from the Registry for Catastrophic Illness, the National Health Insurance Research Database (NHIRD), and the Death Registry Database in Taiwan were analyzed. The NHIRD contains healthcare data of more than 99% of the population in Taiwan, including both inpatient and outpatient medical records.^16^, ^17^ The NHIRD contained patient information such as diagnosis, drug administration, and examinations. The Institutional Review Board of TCH certified this research (no. TCHIRB‐10709107‐W).

### 2.2 Study subjects

Female subjects 18 years and older with a diagnosis of breast cancer between January 1, 2000, and December 31, 2018, were identified from the Registry for Catastrophic Illness (ICD‐9‐CM and ICD‐10‐CM code for female breast cancer: 174 and C50.x1x, respectively). All the cancer diagnoses recorded in the Registry of Catastrophic Illness were confirmed by pathologists.^18^ The Death Registry Database in Taiwan confirmed cases of death. Study subjects were followed until new‐onset IHD diagnosis, death, or December 31, 2018.

### 2.3 Outcome variables

The incidence of IHD was recognized from the NHIRD. It was defined as the occurrence of more than once in inpatient medical records or more than three times in outpatient medical records (ICD‐9‐CM code, 411–414 except 414.1x and ICD‐10‐CM code I20‐I25 except for I21, I25.3, and I25.4).^19^

### 2.4 Main explanatory variable

Information regarding GnRH agonist prescriptions were gathered from the NHIRD. The total administered daily dose of GnRH agonists was calculated and expressed as the defined daily dose (DDD); 0.134 mg for leuprorelin and triptorelin, and 0.129 mg for goserelin, which was suggested by the Anatomical Therapeutic Chemical Classification/Defined Daily Doses (ATC/DDD) system.^20^

### 2.5 Potential confounders

The potential confounders were age, socioeconomic status, breast cancer therapy, including lumpectomy and radiotherapy, and comorbidities. The socioeconomic status included income level and residence. Income level was categorized as low, intermediate, and high (≤19,200; 19,201 to <40,000; ≥40,000 New Taiwan Dollars [NTD]). Residence was categorized as urban, suburban, and rural. The comorbidities were recognized by the presence of disease diagnosis recorded by the International Classification of Diseases, Ninth Revision, Clinical Modification (ICD‐9‐CM) and ICD‐10‐CM code, including diabetes (ICD‐9‐CM:250, ICD‐10‐CM: E08‐E13), chronic kidney disease (ICD‐9‐CM: 585–586, ICD‐10‐CM: N18), hypertension (ICD‐9‐CM: 401–405, ICD‐10‐CM: I1), dyslipidemia (ICD‐9‐CM: 272.0–272.4, ICD‐10‐CM: E78.0‐E78.5), cerebrovascular disease (ICD‐9‐CM: 430–437, ICD‐10‐CM: G46.3‐G46.4, I60‐I66, I69), chronic obstructive pulmonary disease (ICD‐9‐CM: 491–492, 518.1–518.2, 770.2; ICD‐10‐CM: J41‐J44), and liver cirrhosis (ICD‐9‐CM: 491–492, 518.1–518.2, 770.2; ICD‐10‐CM: J41‐J44). Comorbidities were recognized only if the condition occurred more than once in an inpatient setting or more than three times in outpatient medical records.^21^

## 3 STATISTICAL ANALYSIS

First, the demographic data of the study subjects were shown as continuous data with mean and standard deviation (SD) or categorical data with numbers and percentages. Patients with and without GnRH agonist treatment were compared using the two‐sample *t*‐test and Pearson *χ* ^2^ test. The incidence of IHD was calculated using events per 1000 person‐years. Kaplan–Meier survival curves were drawn to show the difference between patients treated with and without GnRH agonists. The Cox regression analysis was used to calculate hazard ratios (HRs) and 95% confidence intervals (CIs). Dose–response relations were also evaluated between GnRH agonist (as a continuous variable) and incident IHD. Death events were analyzed as competing risk events.^22^ Stratified analyses were performed according to age and comorbidities in case interaction may exist. Sensitivity analysis was performed by excluding missing data of the stage of breast cancer and including cancer stage in multivariable Cox regression analysis. The data analyses were conducted using the SAS 9.4 software package (SAS Institute).

## 4 RESULTS

A total of 196,539 female patients with breast cancer were recognized from the Registry for Catastrophic Illness between January 1, 2000, and December 31, 2018. After excluding those with antecedent IHD (*n* = 22,687), younger than 18 years old (*n* = 15), and those with incomplete data (*n* = 987), there were 172,850 patients included in the analysis. Table 1 shows the baseline features of participants. The overall mean (SD) age was 52.6 (11.5) years, and 3.5% of the subjects received treatment with GnRH agonist. The mean (SD) of the DDDs for GnRH agonists was 41.5 (6.4) among patients receiving hormone treatment. Moreover, the mean (SD) follow‐up times were 4.98 (3.80) years in patients receiving GnRH agonists and 7.19 (5.63) years in those not receiving GnRH agonists. Compared with patients not receiving GnRH agonists, those receiving GnRH agonists were younger and more likely to receive lumpectomy and radiotherapy. Moreover, patients receiving GnRH agonists had a lower proportion of comorbidities. Patients received treatment without GnRH agonists were more likely to live in rural areas and have lower incomes.

**TABLE 1:** Characteristics of female patients with breast cancer using GnRH agonists

Characteristics	Total, *n* = 172,850 No. (%) of subjects	Treatment with GnRH agonists, *n* = 6017	Treatment without GnRH agonists, *n* = 166,833	*p*‐Value
Age (years)				
Mean ± SD	52.56 ± 11.47	41.45 ± 6.42	52.96 ± 11.41	<0.001
18–49	75,146 (43.47)	5502 (91.44)	69,644 (41.74)	<0.001
≥50	97,704 (56.53)	515 (8.56)	97,189 (58.26)	
Income level				
Low	18,497 (10.70)	316 (5.25)	18,181 (10.90)	<0.001
Intermediate	65,788 (38.06)	2348 (39.02)	63,440 (38.03)	
High	88,565 (51.24)	3353 (55.73)	85,212 (51.08)	
Urbanization				
Rural	8942 (5.17)	247 (4.11)	8695 (5.21)	<0.001
Suburban	100,028 (57.87)	3515 (58.42)	96,513 (57.85)	
Urban	63,880 (36.96)	2255 (37.48)	61,625 (36.94)	
Lumpectomy				
No	46,731 (27.04)	1243 (20.66)	45,488 (27.27)	<0.001
Yes	126,119 (72.96)	4774 (79.34)	121,345 (72.73)	
Radiotherapy				
No	151,702 (87.77)	4718 (78.41)	146,984 (88.10)	<0.001
Yes	21,148 (12.23)	1299 (21.59)	19,849 (11.90)	
Comorbidity				
Diabetes	37,657 (21.79)	460 (7.65)	37,197 (22.30)	<0.001
Chronic kidney disease	7209 (4.17)	71 (1.18)	7138 (4.28)	<0.001
Hypertension	62,597 (36.21)	710 (11.80)	61,887 (37.10)	<0.001
Dyslipidemia	57,083 (33.02)	759 (12.61)	56,324 (33.76)	<0.001
Cerebrovascular disease	14,812 (8.57)	115 (1.91)	14,697 (8.81)	<0.001
Chronic obstructive pulmonary disease	17,260 (9.99)	293 (4.87)	16,967 (10.17)	<0.001
Liver cirrhosis	34,802 (20.13)	746 (12.40)	34,056 (20.41)	<0.001
Outcomes				
New‐onset of ischemic heart disease	12,605 (7.29)	63 (1.05)	12,542 (7.52)	<0.001
Incidence of ischemic heart disease*	10.24	2.10	10.46	<0.001
Follow‐up years, mean ± SD	7.12 ± 5.59	4.98 ± 3.80	7.19 ± 5.63	<0.001

Abbreviations: GnRH, gonadotropin‐releasing hormone; SD, standard deviation.

* Events per 1000 person‐years.

**FIGURE 1:**
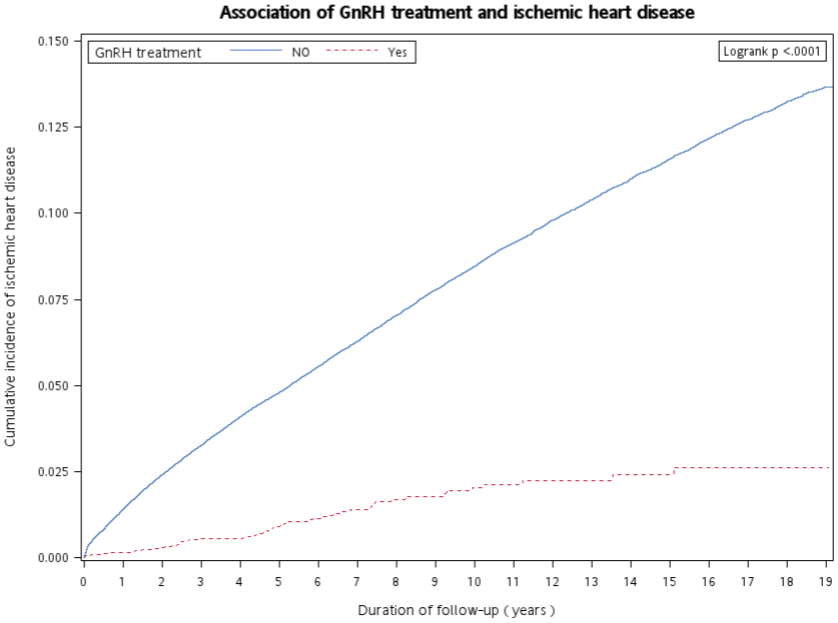
Kaplan–Meier curves for time to diagnosis of incident ischemic heart disease in patients receiving and not receiving GnRH agonists. GnRH, gonadotropin‐releasing hormone.

During the study follow‐up period, 12,605 female patients with breast cancer had a new‐onset of IHD, including 63 (1.05%) patients receiving GnRH agonists and 12,542 (7.52%) patients not receiving GnRH agonists. The incidence rate of IHD per 1000 person‐years was 2.10 in patients receiving GnRH agonists and 10.46 in those not receiving GnRH agonists (*p* < 0.001). In addition, the time to incident IHD was significantly longer in patients receiving GnRH agonists than in those not receiving GnRH agonists (*p* < 0.001, log‐rank test; Figure 1).

**FIGURE 2:**
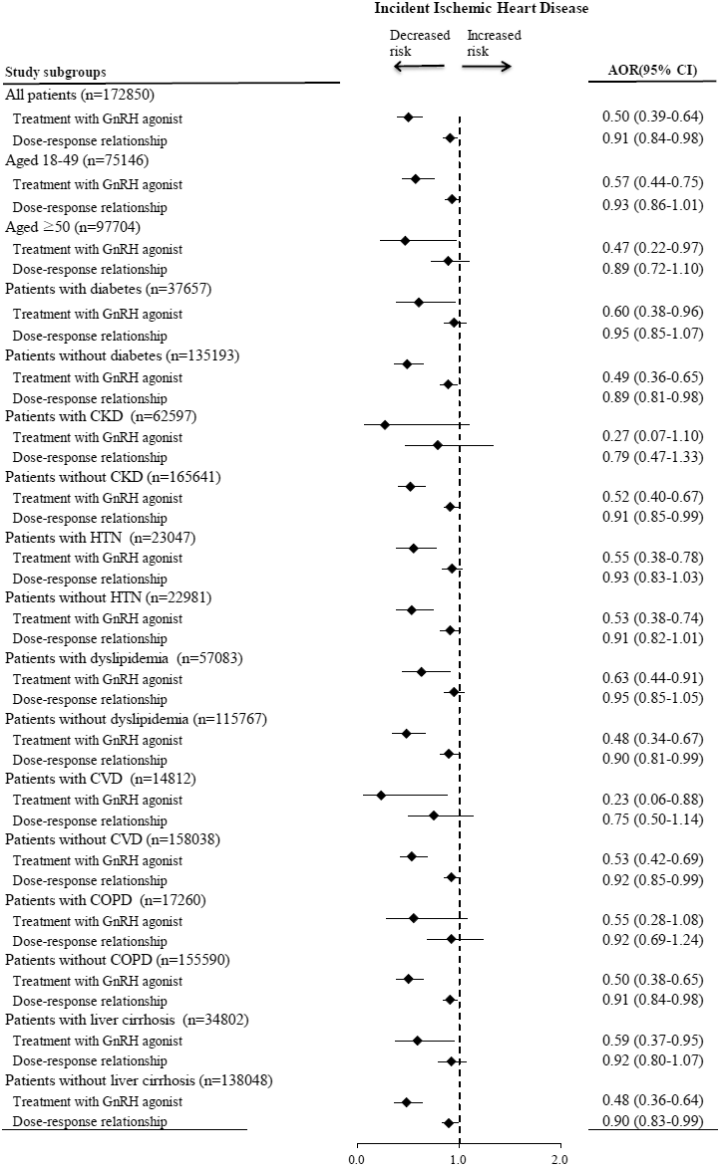
Stratified analysis for the associations of GnRH agonist with incident ischemic heart disease after adjusting for patient characteristics. Values greater than 1.0 indicate increased risk. AHR, adjusted hazard ratio; GnRH, gonadotropin‐releasing hormone.

The univariable Cox proportional hazards model showed that female patients with breast cancer undergoing GnRH agonist therapy had a significantly decreased risk of incident IHD (HR: 0.18, 95% CI: 0.14–0.23). After adjusting for age, sex, and comorbidities, patients using GnRH agonist therapy still had a significantly lower risk of incident IHD (AHR: 0.50; 95% CI: 0.39–0.64) (Table 2). Patients with higher income levels had a lower risk of incident IHD. Other factors associated with decreased risk of incident IHD consisted of lumpectomy and radiotherapy. Moreover, risk factors of incident IHD consisted of age ≥ 50 years, diabetes, chronic kidney disease (CKD), hypertension, dyslipidemia, cerebrovascular disease, chronic obstructive pulmonary disease (COPD), and liver cirrhosis. A significantly linear dose–response effect per DDD increase in GnRH agonists for incident IHD (AHR, 0.91; 95% CI <0.84–0.98; *p* = 0.011) was also noted. Figure 2 showed the results of stratified analysis. GnRH agonists were significantly associated with a lower risk of incident IHD in all the subgroups, except in those with CKD or COPD, respectively. Sensitivity analysis was performed after adjustment for the stage of breast cancer. Patients with missing data of stage were excluded from the analysis(*n* = 104,726). There were 68,124 participants included in multivariable Cox regression analysis. After adjusting for stage of breast cancer, the result showed that female patients with breast cancer undergoing GnRH agonist therapy had a significantly decreased risk of incident IHD (HR: 0.57, 95% CI: 0.38–0.84, *p* = 0.004) (Table S1).

**TABLE 2:** Univariates and multivariate analyses for risk factors associated with ischemic heart disease among patients with breast cancer

Characteristic	Number of patients	Incident IHD	Follow‐up person‐years	Incidence^a^	Univariate analysis HR (95% CI)	Multivariate analysis^b^ AHR (95% CI)
Treatment with GnRH agonist						
No	166,833	12,542	1199529.27	10.46	Ref	Ref
Yes	6017	63	29964.66	2.10	0.18 (0.14–0.23)*	0.50 (0.39–0.64)*
Age (years)						
18–49	75,146	2960	620705.96	4.77	Ref	Ref
≥50	97,704	9645	609672.96	15.82	2.96 (2.84–3.08)*	1.43 (1.37–1.50)*
Income level						
Low	18,497	2012	107652.54	18.69	Ref	Ref
Intermediate	65,788	4598	453937.20	10.13	0.71 (0.67–0.75)*	0.88 (0.83–0.92)*
High	88,565	5995	668665.75	8.97	0.68 (0.65–0.72)*	0.90 (0.85–0.94)*
Urbanization						
Rural	8942	846	61252.70	13.81	Ref	Ref
Suburban	100,028	7058	708198.24	9.97	0.76 (0.70–0.81)*	0.87 (0.81–0.94)*
Urban	63,880	4701	461213.60	10.19	0.79 (0.73–0.85)*	0.93 (0.86–1.00)*
Lumpectomy						
No	46,731	5809	368240.28	15.78	Ref	Ref
Yes	126,119	6796	862653.96	7.88	0.53 (0.51–0.55)*	0.61 (0.58–0.63)*
Radiotherapy						
No	151,702	11,685	1095288.44	10.67	Ref	Ref
Yes	21,148	920	134501.28	6.84	0.64 (0.60–0.68)*	0.89 (0.84–0.96)*
Diabetes						
No	135,193	6796	939591.35	7.23	Ref	Ref
Yes	37,657	5809	289958.90	20.03	2.90 (2.80–3.00)*	1.17 (1.12–1.21)*
Chronic kidney disease						
No	165,641	10,855	1176051.10	9.23	Ref	Ref
Yes	7209	1750	54355.86	32.20	3.53 (3.36–3.72)*	1.55 (1.47–1.63)*
Hypertension						
No	110,253	2782	755233.05	3.68	Ref	Ref
Yes	62,597	9823	474485.26	20.70	5.91 (5.66–6.16)*	3.19 (3.04–3.35)*
Dyslipidemia						
No	115,767	4557	765219.87	5.96	Ref	Ref
Yes	57,083	8048	464655.62	17.32	3.42 (3.30–3.55)*	1.77 (1.70–1.85)*
Cerebrovascular disease						
No	158,038	9375	1118909.04	8.38	Ref	Ref
Yes	14,812	3230	110764.14	29.16	3.43 (3.30–3.57)*	1.56 (1.49–1.63)*
Chronic obstructive pulmonary disease						
No	155,590	9759	1095353.60	8.91	Ref	Ref
Yes	17,260	2846	134628.00	21.14	2.47 (2.37–2.57)*	1.57 (1.50–1.64)*
Liver cirrhosis						
No	13,8048	8534	949770.24	8.99	Ref	Ref
Yes	34,802	4071	280504.12	14.51	1.73 (1.67–1.80)*	1.22 (1.17–1.26)*

Abbreviations: AHR, adjusted hazard ratio; CI, confident interval; GnRH, gonadotropin‐releasing hormone; HR, hazard ratio; IHD, ischemic heart disease.

^a^ Events per 1000 person‐years.

^b^ Adjusted for: age, income level, urbanization, lumpectomy, radiotherapy, and comorbidities (diabetes, chronic kidney disease, hypertension, dyslipidemia, cerebrovascular disease, chronic obstructive pulmonary disease, and liver cirrhosis).

* <0.001.

## 5 DISCUSSION

This study found that female patients with breast cancer receiving GnRH agonists had a lower risk of developing IHD than patients not receiving GnRH agonists.

GnRH agonists bind to GnRH receptors in the pituitary gland, resulting in the secretion and initial surge of FSH and LH which stimulates the production of serum testosterone or estrogen. Subsequently, the negative feedback at the pituitary gland causes downregulation of GnRH receptors. On the contrary, no initial testosterone surge is found after administration of GnRH antagnosits.^14^ The distinct impact of GnRH agonists in our study, and bilateral oophorectomy on IHD, might be partially explained by the fact that serum FSH and LH is sustainably inhibited after GnRH agonist administration but upregulated after bilateral oophorectomy.^23^ Potential alternative mechanisms explaining the findings of our study were adipogenesis^24^ and atherosclerosis.^25^ Dysregulated fat deposits to the arterial wall cause atherosclerosis and IHD.^26^ Peripheral blood mononuclear cells (PMN) and pro‐inflammatory T helper 1 lymphocytes both express GnRH receptors. The activation of these receptors is involved in the activation of PMNs, lymphocytes, and cytokine production, such as an increase in IFN‐γ, and decrease in IL‐4.^27^, ^28^ Different effects of GnRH‐I and GnRH‐II demonstrated that GnRH‐I enhanced proliferation of PMNs and IL‐2Rγ expression, while GnRH‐II attenuated proliferation of PMNs and IL‐2Rγ expression.^29^

A large population study evaluating the side effects of bilateral oophorectomy‐induced menopause on premenopausal women before age 50 without hormone replacement therapy (HRT) demonstrated a statistically significant increased risk of multimorbidity including hyperlipidemia, and diabetes mellitus. The side effects of coronary artery disease became statistically significant only in adjusted analyses restricted to females receiving oophorectomy before the age of 45.^23^, ^30^ The deleterious effects of natural estrogen deprivation after menopause in the Study of Women's Health Across the Nation (SWAN) comprises of increased body and cardiovascular fat and alternations in body weight and waist circumference.^31^, ^32^, ^33^ Association between lumpectomy and IHD risk was not yet investigated in previous studies. The procedure of lumpectomy may not be associated with pathogenesis of IHD. In this study, we tried to included detailed treatment procedure, including surgical procedure, radiotherapy, and medical treatment. The detailed surgical procedure was not available in our dataset. Further research is warranted to explore impact of lumpectomy on IHD risk. Previous studies had demonstrated that exposure of the heart to ionizing radiation during radiotherapy for breast cancer increases the subsequent rate of ischemic heart disease.^34^ But the results of this study showed that radiotherapy appeared to be associated with lower risk of IHD. The detailed radiation therapy regimen including dose and area were not available in this dataset. Even radiotherapy for distal bone metastasis were included in analysis, which may lead to bias on IHD risks of radiotherapy.

This study enrolled a large number of patients with breast cancer and had a long follow duration from 2000 to 2018. The diagnoses of breast cancer were confirmed by pathology reports in the Registry for Catastrophic Illness, and the diagnoses of comorbidities were confirmed by medical reports to ensure the validity of this study. Additionally, socioeconomic status and treatment strategies were included as potential confounders. Our study has several limitations. First, similarly to other retrospective population studies, patients were not randomized to both treatment groups. Patients allocated to the GnRH treatment group had significantly higher income levels, urbanization, more lumpectomy, and radiotherapy. However, these patients were younger and had fewer comorbidities including diabetes mellitus and dyslipidemia. Nonetheless, multivariate analysis demonstrated treatment with GnRH agonists as an independent predictive factor associated with lower risk of IHD. The stratified analysis also showed that GnRH agonists were significantly associated with a lower risk of IHD in all subgroups of patients. Second, we used ICD codes to identify the diagnosis of IHD in the administrative database. Although patients with less frequent visits were less likely to be diagnosed with IHD, the frequency of visits ranged from once every month to every 3 months. Patients receiving GnRH agonists usually received treatment at a one‐month interval, which made the attribution of lower risk of IHD to lower frequency of visits less likely. The generalizability of this study to other regions requires further certification because most of the study subjects were Taiwanese.

Our study provides preliminary report for evaluating breast cancer treatment, considering the scarce literature currently available regarding the associations of GnRH agonists and the risk of IHD among women with breast cancer. In conclusion, our large population study is the first to report that treatment using GnRH agonists for patients with breast cancer was associated with a significantly reduced risk of IHD after adjusting for variable confounders. Furthermore, endocrine therapy for breast cancer treatment should weigh the benefits of disease‐specific survival against long‐term side effects of cardiovascular events. Patients receiving endocrine therapy should try to avoid risk factors of cardiovascular disease. Further research to delineate and confirm the causality and mechanisms is needed.

## AUTHOR CONTRIBUTIONS

**Yi‐Sheng Chou:** Conceptualization (lead); writing – original draft (lead). **Chun‐Chieh Wang:** Investigation (equal). **Li‐Fei Hsu:** Data curation (equal); investigation (equal). **Pei‐Hung Chuang:** Data curation (equal); formal analysis (equal). **Chi‐Feng Cheng:** Investigation (equal). **Nai‐Hsin Li:** Conceptualization (equal); investigation (equal). **Chu‐Chieh Chen:** Supervision (equal); visualization (equal). **Chien‐Liang Chen:** Conceptualization (equal); supervision (equal). **Yun‐Ju Lai:** Funding acquisition (equal); investigation (equal); resources (equal); visualization (equal); writing – review and editing (equal). **Yung‐Feng Yen:** Conceptualization (equal); data curation (equal); formal analysis (equal); investigation (equal); writing – original draft (equal); writing – review and editing (equal).

## FUNDING INFORMATION

This research is supported by Taichung Veterans General Hospital of Puli branch, Grant Number: PL‐2021002.

## CONFLICT OF INTEREST

The authors declare no competing interests.

## Supporting information


Table S1

## Data Availability

The datasets of the current study are available from the Health and Welfare Data Science Center of Ministry of Health and Welfare in Taiwan.
