# Supplementary material for: Gonadotropin‐releasing hormone agonist treatment and ischemic heart disease among female patients with breast cancer: A cohort study
Source: Cancer Med. 2022 Oct 28;12(5):5536–44. doi: 10.1002/cam4.5390 (PMC10028063; doi:10.1002/cam4.5390)
Supplement: Supplementary file 1 — Table S1 [file CAM4-12-5536-s001.docx]

Table 1. Characteristics of female patients with breast cancer using GnRH agonists

| Characteristics | Total, n=172,850  No. (%) of subjects | Treatment with GnRH agonists, n=6,017 | Treatment without GnRH agonists, n=166,833 | P-value |
| --- | --- | --- | --- | --- |
| Age (years)  Mean ± SD  18-49  ≥50 | 52.56 ± 11.47  75,146 (43.47)  97,704 (56.53) | 41.45 ± 6.42  5,502 (91.44)  515 (8.56) | 52.96 ± 11.41  69,644 (41.74)  97,189 (58.26) | <.001  <.001 |
| Income level  Low  Intermediate  High | 18,497 (10.70)  65,788 (38.06)  88,565 (51.24) | 316 (5.25)  2,348 (39.02)  3,353 (55.73) | 18,181 (10.90)  63,440 (38.03)  85,212 (51.08) | <.001 |
| Urbanization  Rural  Suburban  Urban | 8,942 (5.17)  100,028 (57.87)  63,880 (36.96) | 247 (4.11)  3,515 (58.42)  2,255 (37.48) | 8,695 (5.21)  96,513 (57.85)  61,625 (36.94) | <.001 |
| Lumpectomy  No  Yes | 46,731 (27.04)  126,119 (72.96) | 1,243 (20.66)  4,774 (79.34) | 45,488 (27.27)  121,345 (72.73) | <.001 |
| Radiotherapy  No  Yes | 151,702 (87.77)  21,148 (12.23) | 4,718 (78.41)  1,299 (21.59) | 146,984 (88.10)  19,849 (11.90) | <.001 |
| Comorbidity  Diabetes  Chronic kidney disease  Hypertension  Dyslipidemia  Cerebrovascular disease  Chronic obstructive pulmonary disease  Liver cirrhosis | 37,657 (21.79)  7,209 (4.17)  62,597 (36.21)  57,083 (33.02)  14,812 (8.57)  17,260 (9.99)  34,802 (20.13) | 460 (7.65)  71 (1.18)  710 (11.80)  759 (12.61)  115 (1.91)  293 (4.87)  746 (12.40) | 37,197 (22.30)  7,138 (4.28)  61,887 (37.10)  56,324 (33.76)  14,697 (8.81)  16,967 (10.17)  34,056 (20.41) | <.001  <.001  <.001  <.001  <.001  <.001  <.001 |
| Outcomes  New onset of ischemic heart disease  Incidence of ischemic heart disease*  Follow-up years, mean ± SD | 12,605 (7.29)  10.24  7.12 ± 5.59 | 63 (1.05)  2.10  4.98 ± 3.80 | 12,542 (7.52)  10.46  7.19 ± 5.63 | <.001  <.001  <.001 |

SD, standard deviation; GnRH, gonadotropin-releasing hormone, *events per 1,000 person-years.

Table 2. Univariates and multivariate analyses for risk factors associated with ischemic heart disease among patients with breast cancer

| Characteristic | Number of patients | Incident IHD | Follow-up person-years | Incidence^a^ | Univariate analysis  HR (95% CI) | Multivariate analysis^b^  AHR (95% CI) |
| --- | --- | --- | --- | --- | --- | --- |
| Treatment with GnRH agonist  No  Yes | 166,833  6,017 | 12,542  63 | 1199529.27  29964.66 | 10.46  2.10 | Ref  0.18 (0.14-0.23)* | Ref  0.50 (0.39-0.64)* |
| Age (years)  18-49  ≥50 | 75,146  97,704 | 2,960  9,645 | 620705.96  609672.96 | 4.77  15.82 | Ref  2.96 (2.84-3.08)* | Ref  1.43 (1.37-1.50)* |
| Income level  Low  Intermediate  High | 18,497  65,788  88,565 | 2,012  4,598  5,995 | 107652.54  453937.20  668665.75 | 18.69  10.13  8.97 | Ref  0.71 (0.67-0.75)*  0.68 (0.65-0.72)* | Ref  0.88 (0.83-0.92)*  0.90 (0.85-0.94)* |
| Urbanization  Rural  Suburban  Urban | 8,942  100,028  63880 | 846  7,058  4,701 | 61252.70  708198.24  461213.60 | 13.81  9.97  10.19 | Ref  0.76 (0.70-0.81)*  0.79 (0.73-0.85)* | Ref  0.87 (0.81-0.94)*  0.93 (0.86-1.00) |
| Lumpectomy  No  Yes | 46,731  126,119 | 5,809  6,796 | 368240.28  862653.96 | 15.78  7.88 | Ref  0.53 (0.51-0.55)* | Ref  0.61 (0.58-0.63)* |
| Radiotherapy  No  Yes | 151,702  21,148 | 11,685  920 | 1095288.44  134501.28 | 10.67  6.84 | Ref  0.64 (0.60-0.68)* | Ref  0.89 (0.84-0.96)* |
| Diabetes  No  Yes | 135,193  37,657 | 6,796  5,809 | 939591.35  289958.90 | 7.23  20.03 | Ref  2.90 (2.80-3.00)* | Ref  1.17 (1.12-1.21)* |
| Chronic kidney disease  No  Yes | 165,641  7,209 | 10,855  1,750 | 1176051.10  54355.86 | 9.23  32.20 | Ref  3.53 (3.36-3.72)* | Ref  1.55 (1.47-1.63)* |
| Hypertension  No  Yes | 110253  62597 | 2,782  9,823 | 755233.05  474485.26 | 3.68  20.70 | Ref  5.91 (5.66-6.16)* | Ref  3.19 (3.04-3.35)* |
| Dyslipidemia  No  Yes | 115,767  57,083 | 4,557  8,048 | 765219.87  464655.62 | 5.96  17.32 | Ref  3.42 (3.30-3.55)* | Ref  1.77 (1.70-1.85)* |
| Cerebrovascular disease  No  Yes | 158,038  14,812 | 9,375  3,230 | 1118909.04  110764.14 | 8.38  29.16 | Ref  3.43 (3.30-3.57)* | Ref  1.56 (1.49-1.63)* |
| Chronic obstructive pulmonary disease  No  Yes | 155,590  17,260 | 9,759  2,846 | 1095353.60  134628.00 | 8.91  21.14 | Ref  2.47 (2.37-2.57)* | Ref  1.57 (1.50-1.64)* |
| Liver cirrhosis  No  Yes | 13,8048  34,802 | 8,534  4,071 | 949770.24  280504.12 | 8.99  14.51 | Ref  1.73 (1.67-1.80)* | Ref  1.22 (1.17-1.26)* |

*<.001

^a^ events per 1,000 person-years.

^b^ Adjusted for: age, income level, urbanization, lumpectomy, radiotherapy, and comorbidities (diabetes, chronic kidney disease, hypertension, dyslipidemia, cerebrovascular disease, chronic obstructive pulmonary disease, and liver cirrhosis).

Abbreviations: GnRH, Gonadotropin-Releasing Hormone; IHD, ischemic heart disease; HR= hazard ratio; AHR=adjusted hazard ratio; CI=confident interval

Supplementary table 1. Sensitivity analysis by excluding missing data of the stage of breast cancer (n=68,124)

| **Characteristic** | **AHR** | **(95% CI)** | **p-value** |
| --- | --- | --- | --- |
| Treatment with GnRH agonist  No  Yes | Ref  0.57 | (0.38-0.84) | 0.004 |
| Age (years)  18-49  ≥50 | Ref  1.43 | (1.29-1.59) | <.001 |
| Income level  Low  Intermediate  High | Ref  0.98  0.90 | (0.85-1.13)  (0.78-1.03) | 0.79  0.13 |
| Urbanization  Rural  Suburban  Urban | Ref  1.03  1.17 | (0.86-1.24)  (0.98-1.41) | 0.72  0.09 |
| Radiotherapy  No  Yes | Ref  0.95 | (0.84-1.07) | 0.37 |
| Diabetes  No  Yes | Ref  1.20 | (1.10-1.31) | <.001 |
| Chronic kidney disease  No  Yes | Ref  1.85 | (1.64-2.09) | <.001 |
| Hypertension  No  Yes | Ref  2.87 | (2.59-3.18) | <.001 |
| Dyslipidemia  No  Yes | Ref  1.71 | (1.56-1.88) | <.001 |
| Cerebrovascular disease  No  Yes | Ref  1.33 | (1.20-1.48) | <.001 |
| Chronic obstructive pulmonary disease  No  Yes | Ref  1.37 | (1.23-1.52) | <.001 |
| Liver cirrhosis  No  Yes | Ref  1.08 | (0.99-1.17) | 0.10 |
| Stage  0  I  II  III  IV | Ref  0.77  0.71  0.62  0.37 | (0.77-0.66)  (0.71-0.61)  (0.62-0.52)  (0.37-0.27) | 0.001  <.001  <.001  <.001 |
